# Supplementary material for: Epidemiology and Comparative Analyses of the S Gene on Feline Coronavirus in Central China
Source: Pathogens. 2022 Apr 12;11(4):460. doi: 10.3390/pathogens11040460 (PMC9031646; doi:10.3390/pathogens11040460)
Supplement: Supplementary file 1 [file pathogens-11-00460-s001.zip › pathogens-1654001-supplementary.pdf]

Table S1 Information related to sequences in the gene bank

No.	Strain name	Collection node	Living environment	Breed	Gender	Age	Sample source	Detection of FCoV based on 3′UTR gene	Genotyping of FCoVs based on partial S gene		Genbank accession No. of partial S genes of FCoVs
									Type Ⅰ of FCoV	Type Ⅱ of FCoV	
1	FIPV/HB/WH/2020/74/f2	Oct-20	/				ascites	+	+		OK236259
2	FCoV/HB/WH/2019/10/Zhuangzhuang	Nov-19	Multi-cat environment	BSH	M	12M	feces	+		+	OK236260
3	FCoV/HB/WH/2019/12/Xiaosongshu	Nov-19	Multi-cat environment	DSH	M	24M	feces	+	+		OK236261
4	FCoV/HB/WH/2019/15/Qinqin	Nov-19	Multi-cat environment	DSH	M	24M	feces	+	+		OK236262
5	FCoV/HB/WH/2019/16/Yuanliuliu	Nov-19	Multi-cat environment	DSH	M	8M	feces	+	+		OK236263
6	FCoV/HB/WH/2019/17/Xiaoxiao	May-19	Multi-cat environment	DSH	M	24M	feces	+	+		OK236264
7	FCoV/HB/WH/2019/19/Doudou	Oct-19	Multi-cat environment	DSH	M	8M	feces	+	+		OK236265
8	FCoV/HB/WH/2019/2/Ali	Mar-19	Multi-cat environment	DSH	F	36M	feces	+	+		OK236266
9	FCoV/HB/WH/2019/20/Huli	Apr-19	Multi-cat environment	DSH	F	24M	feces	+		+	OK236267
10	FCoV/HB/WH/2019/24/Wawa	May-19	Multi-cat environment	BSH	F	10M	feces	+		+	OK236268
11	FCoV/HB/WH/2019/31/Aobai	May-19	Single cat household	ELH	F	7M	feces	+	+		OK236269
12	FCoV/HB/WH/2019/33/Erhao	Jun-19	Multi-cat environment	ESH	M	3M	feces	+	+		OK236270
13	FCoV/HB/WH/2019/34/Sanhao	Jun-19	Multi-cat environment	ESH	F	3M	feces	+	+		OK236271
14	FCoV/HB/WH/2019/38/Xiaoliu	Jul-19	Multi-cat environment	BSH	F	10M	feces	+	+		OK236272
15	FCoV/HB/WH/2019/4/Lugouqiao	Mar-19	Multi-cat environment	DSH	F	24M	feces	+	+		OK236273

16	FCoV/HB/WH/2019/43/Miantuan	Jul-19	Multi-cat environment	DSH	M	60M	feces	+	+		OK236274
17	FCoV/HB/WH/2019/5/Jianjian	Mar-19	Multi-cat environment	DSH	F	36M	feces	+	+		OK236275
18	FCoV/HB/WH/2019/50/Dahong	Jul-19	Multi-cat environment	BSH	F	33M	feces	+	+		OK236276
19	FCoV/HB/WH/2019/51/Meimei	Jun-19	Multi-cat environment	BSH	F	33M	feces	+	+		OK236277
20	FCoV/HB/WH/2019/52/Momo	Jul-19	Multi-cat environment	BSH	F	3M	feces	+	+		OK236278
21	FCoV/HB/WH/2019/53/Danxiaogui	Aug-19	Multi-cat environment	BSH	M	8M	feces	+	+		OK236279
22	FCoV/HB/WH/2019/54/Dada	Aug-19	Multi-cat environment	BSH	M	8M	feces	+	+		OK236280
23	FCoV/HB/WH/2019/55/Pangpang	Sep-19	Multi-cat environment	BSH	M	7M	feces	+	+		OK236281
24	FCoV/HB/WH/2019/56/Taotao	Sep-19	Multi-cat environment	BSH	F	7M	feces	+	+		OK236282
25	FCoV/HB/WH/2019/57/Zhuangzhuang2	Sep-19	Multi-cat environment	BSH	F	7M	feces	+	+		OK236283
26	FCoV/HB/WH/2019/58/Wawa2	Oct-19	Multi-cat environment	BSH	F	7M	feces	+	+		OK236284
27	FCoV/HB/WH/2019/59/Hei	Oct-19	Multi-cat environment	DSH	M	6M	feces	+	+		OK236285
28	FCoV/HB/WH/2019/6/Xiaohong	Mar-19	Multi-cat environment	BSH	F	10M	feces	+	+		OK236286
29	FCoV/HB/WH/2019/64/Dazhuang	Nov-19	Multi-cat environment	BSH	M	24M	feces	+		+	OK236287
30	FCoV/HB/WH/2019/66/Shan	Nov-19	Multi-cat environment	ESH	F	24M	feces	+	+		OK236288
31	FCoV/HB/WH/2019/7/Lvyan	Apr-19	Multi-cat environment	DSH	M	24M	feces	+	+		OK236289
32	FCoV/HB/WH/2019/8/Xiaohui	Apr-19	Multi-cat environment	BSH	M	10M	feces	+		+	OK236290

33	FIPV/HB/WH/2019/1/Chami	Mar-19	/	ELH	M	7M	ascites	+	+	OK236291
34	FIPV/HB/WH/2019/11/Runtu	Mar-19	Multi-cat environment	BSH	M	6M	ascites	+	+	OK236292
35	FIPV/HB/WH/2019/15/Latiao	May-19	Multi-cat environment	BSH	M	7M	ascites	+	+	OK236293
36	FIPV/HB/WH/2019/16/Baozi	May-19	Multi-cat environment	BSH	F	4M	ascites	+	+	OK236294
37	FIPV/HB/WH/2019/18/Jiujiu	May-19	Single cat household	BSH	F	3M	ascites	+	+	OK236295
38	FIPV/HB/WH/2019/20/Shiqi	May-19	Single cat household	ESH	M	6M	ascites	+	+	OK236296
39	FIPV/HB/WH/2019/21/Zhanan	Jun-19	Single cat household	BSH	M	9M	ascites	+	+	OK236297
40	FIPV/HB/WH/2019/25/Heiya	Sep-19	/	DSH	F	4M	ascites	+	+	OK236298
41	FIPV/HB/WH/2019/28/Kele	Nov-19	Single cat household	ASH	M	7M	ascites	+	+	OK236299
42	FIPV/HB/WH/2019/3/Maodou	Feb-19	Multi-cat environment	ESH	M	8M	ascites	+	+	OK236300
43	FIPV/HB/WH/2019/38/Wanwan	Dec-19	/	BSH	F	6M	ascites	+	+	OK236301
44	FIPV/HB/WH/2019/41/Nini	Aug-19	Single cat household	BSH	M	5M	ascites	+	+	OK236302
45	FIPV/HB/WH/2019/47/Luna	Dec-19	Multi-cat environment	DSH	F	18M	ascites	+	+	OK236303
46	FIPV/HB/WH/2019/48/Wubai	Dec-19	Multi-cat environment	BSH	F	4M	ascites	+	+	OK236304
47	FIPV/HB/WH/2019/8/Hanhan	Dec-19	/	ELH	F	14M	ascites	+	+	OK236305
48	FCoV/HB/WH/2020/107/Wanzi	Sep-20	Single cat household	DSH	F	5M	feces	+	+	OK236306
49	FCoV/HB/WH/2020/108/107	Sep-20	Single cat household	DSH	F	5M	feces	+	+	OK236307
50	FCoV/HB/WH/2020/126/Nomi	Oct-20	Single cat household	BSH	M	12M	feces	+	+	OK236308
51	FCoV/HB/WH/2020/134/Naiqiu	Oct-20	Single cat household	ELH	M	5M	feces	+	+	OK236309

52	FCoV/HB/WH/2020/136/Babao	Oct-20	Single cat household	ESH	M	12M	feces	+	+	OK236310
53	FCoV/HB/WH/2020/141/Tiantian	Oct-20	Single cat household	BSH	F	2M	feces	+	+	OK236311
54	FCoV/HB/WH/2020/143/youzi	Oct-20	Single cat household	ELH	F	3M	feces	+	+	OK236312
55	FCoV/HB/WH/2020/145/Rourou	Oct-20	Single cat household	BSH	F	29M	feces	+	+	OK236313
56	FCoV/HB/WH/2020/147/Libai	Oct-20	Single cat household	ESH	M	48M	feces	+	+	OK236314
57	FCoV/HB/WH/2020/149/Huasheng	Oct-20	Single cat household	BSH	F	16M	feces	+	+	OK236315
58	FCoV/HB/WH/2020/151/Anni	Nov-20	Single cat household	ESH	F	36M	feces	+	+	OK236316
59	FCoV/HB/WH/2020/153/Dali	Nov-20	Multi-cat environment	BSH	M	2M	feces	+	+	OK236317
60	FCoV/HB/WH/2020/154/Sifeng	Nov-20	Single cat household	/	M	3M	feces	+	+	OK236318
61	FCoV/HB/WH/2020/156/Dagou	Nov-20	Single cat household	BSH	M	6M	feces	+	+	OK236319
62	FCoV/HB/WH/2020/164/Paofu	Nov-20	Single cat household	ELH	F	4M	feces	+	+	OK236320
63	FCoV/HB/WH/2020/188/Chuchu	Dec-20	Single cat household	DSH	M	3M	feces	+	+	OK236321
64	FCoV/HB/WH/2020/193/Mengmeng	Sep-20	Multi-cat environment	DSH	F	2M	feces	+	+	OK236322
65	FCoV/HB/WH/2020/195/Susu	Sep-20	Multi-cat environment	ESH	F	16M	feces	+	+	OK236323
66	FCoV/HB/WH/2020/198/lucky	Sep-20	Multi-cat environment	ESH	M	12M	feces	+	+	OK236324
67	FCoV/HB/WH/2020/200/Kafei	Sep-20	Multi-cat environment	ESH	M	32M	feces	+	+	OK236325
68	FCoV/HB/WH/2020/201/kaka	Sep-20	Multi-cat environment	ESH	M	28M	feces	+	+	OK236326

69	FCoV/HB/WH/2020/205/Xiangxiang	Sep-20	Multi-cat environment	ESH	F	12M	feces	+	+	OK236327
70	FCoV/HB/WH/2020/206/xueshang	Sep-20	Multi-cat environment	ESH	M	28M	feces	+	+	OK236328
71	FCoV/HB/WH/2020/212/Niangao	Sep-20	Multi-cat environment				feces	+	+	OK236329
72	FCoV/HB/WH/2020/224/Mimi	Nov-20	Multi-cat environment	ESH	F	7M	feces	+	+	OK236330
73	FCoV/HB/WH/2020/226/qiuqiu	Dec-20	Single cat household		M	3M	feces	+	+	OK236331
74	FCoV/HB/WH/2020/230/changshou	Sep-20	Single cat household	ELH	F	4W	feces	+	+	OK236332
75	FCoV/HB/WH/2020/232/Baixiaobai	Sep-20	Single cat household	DSH	M	3M	feces	+	+	OK236333
76	FCoV/HB/WH/2020/235/Fugui	Oct-20	Single cat household	DSH	F	7M	feces	+	+	OK236334
77	FCoV/HB/WH/2020/238/Tuanzi	Nov-20	Single cat household	BSH	M	6M	feces	+	+	OK236335
78	FCoV/HB/WH/2020/242/Doubao	Jan-20	Single cat household	ESH	F	4M	feces	+	+	OK236336
79	FCoV/HB/WH/2020/249/Daliu	Dec-20	Multi-cat environment	DSH			feces	+	+	OK236337
80	FCoV/HB/WH/2020/250/Juzi	Dec-20	/	DSH	M	24M	feces	+	+	OK236338
81	FCoV/HB/WH/2020/77/16	Aug-20	/	ESH	M	8M	feces	+	+	OK236339
82	FCoV/HB/WH/2020/78/18	Aug-20	/	ELH	M	9M	feces	+	+	OK236340
83	FCoV/HB/WH/2020/82/28	Dec-20	/				feces	+	+	OK236341
84	FIPV/HB/WH/2020/51/huahua	Dec-20	Single cat household	ASH	M	17M	ascites	+	+	OK236342
85	FIPV/HB/WH/2020/73/Xiaoba	Oct-20	/				ascites	+	+	OK236343

Notes：For breed, ASH: American Shorthair; BSH: British Shorthair; DSH: Domestic Shorthair; ESH: Exotic Shorthair; ELH: Exotic Longhair. For gender, F: female, and M: male. For age, M: month. NA: not available. For sample source, CHC: clinically healthy cat; FSC: FIP-suspected cat. "+" represents positive results of viral detection; "–"represents negative results of viral detection

Table S2 Correlation of FCoV prevalence with clinical status, sex, breed, age and residential density

	Total number of samples	FCoV positive number	Positive rate	FCoV negative number	Negative rate	χ^2	P	OR	95%CI
	371	172	46.4%	199	53.6%				
**Clinical status**	n=371					6.932	0.008		
FIP suspected cat	81	48	59.3%	33	40.7%			1.683	1.135-2.494
Non-FIP cat	290	124	42.8%	166	57.2%			0.864	0.773-0.966
**Gender**	n=358					0.634	0.426		
male	186	90	48.4%	96	51.6%			1.084	0.889-1.323
female	172	76	44.2%	96	55.8%			0.916	0.736-1.138
**Variety**	n=351					7.901	0.005		
Mongrel cat	127	46	36.2%	81	63.8%			0.663	0.493-0.890
Purebred cat	224	116	51.8%	108	48.2%			1.253	1.071-1.466
**Age**	n=361					5.953	0.015		
≤10M	211	109	51.7%	102	48.3%			1.241	1.044-1.477
A>10M	150	58	38.7%	92	61.3%			0.732	0.567-0.945
**Residential density**	n=323					12.613	0.000		
Single	218	81	37.2%	137	62.8%			0.754	0.639-0.889
Multiple	105	61	58.1%	44	41.9%			1.767	1.284-2.432

In the process of collecting clinical information, some pet owners did not disclose some pet-related information, resulting in a different total sample volume when analyzing each risk factor.

χ2: Chi-square; OR: Odds Ratio; C: Confidence Interval; M: Months.

Table S3 The relationship between FCoV and diarrhea in Non-FIP cat

Symptom	Total number of sample	FCoV positive	FCoV negative	χ^2	p	OR	95%CI
	n=290			0.395	0.530		
Diarrhea	116	53（42.1%）	63（38.4%）			1.095	0.826-1.452
Non-diarrhea	174	73（57.9%）	101（61.6%）			0.941	0.777-1.140

Table S4 The reference sequence involved and its related information

NCBI No.	Country	Collection time	Where the reference sequence is used in the text			Virus
NC045512.2	China	2019	/	Phylogenetics	/	2019-nCoV
NC004718	Canada	2003	/	Phylogenetics	/	SARS-CoV
MZ724506.1	India	2021	/	Phylogenetics	/	SARS-CoV-2
KC164505.2	UK	2012	/	Phylogenetics	/	MERS-CoV
KP981644	Italy	2005	/	Phylogenetics	/	CCoV
NC038861	USA	2000	/	Phylogenetics	/	TEGV
NC002306.3	USA	2005	Mutation	Phylogenetics	/	FIPV
MW030110.1	USA	2002	Mutation	Phylogenetics	Furin cleavage	FCoV
MW030109.1	USA	2018	Mutation	/	/	FIPV
MW030108.1	China	2021	Mutation	Phylogenetics	Furin cleavage	FCoV
MT444152.1	China	2019	Mutation	Phylogenetics	Furin cleavage	FIPV
MT239440.1	China	2017	Mutation	Phylogenetics	/	FCoV
MT239439.1	China	2016	Mutation	Phylogenetics	Furin cleavage	FCoV
MN165107.1	China	2018	Mutation	Phylogenetics	Furin cleavage	FCoV
MG893511.1	Germany	2012	Mutation	Phylogenetics	Furin cleavage	FIPV
KY566211.1	China	2016	Mutation	Phylogenetics	Furin cleavage	FIPV
KY566210.1	China	2016	Mutation	Phylogenetics	Furin cleavage	FIPV
KY566209.1	China	2016	Mutation	Phylogenetics	Furin cleavage	FIPV
KY292377.1	China	2016	Mutation	Phylogenetics	Furin cleavage	FIPV
KX722530.1	Denmark	2015	Mutation	Phylogenetics	Furin cleavage	FIPV
KX722529.1	Belgium	2015	Mutation	Phylogenetics	Furin cleavage	FCoV
KU215419.1	Belgium	2013	Mutation	Phylogenetics	Furin cleavage	FIPV
KP143512.1	UK	2013	Mutation	Phylogenetics	Furin cleavage	FIPV
KP143511.1	UK	2013	Mutation	/	Furin cleavage	FCoV
KP143510.1	UK	2013	Mutation	/	Furin cleavage	FCoV
KP143509.1	UK	2013	Mutation	/	Furin cleavage	FCoV
KP143508.1	UK	2013	Mutation	Phylogenetics	Furin cleavage	FIPV
KP143507.1	UK	2013	Mutation	Phylogenetics	Furin cleavage	FIPV
KF530123.1	NLD	2010	Mutation	Phylogenetics	/	FCoV
JN634064.1	USA	2011	Mutation	Phylogenetics	/	FCoV

JN183883.1	NLD	2010	Mutation	Phylogenetics	Furin cleavage	FCoV
JN183882.1	NLD	2010	Mutation	Phylogenetics	Furin cleavage	FCoV
HQ392472.1	NLD	2010	Mutation	Phylogenetics	Furin cleavage	FCoV
HQ392471.1	NLD	2007	Mutation		Furin cleavage	FCoV
HQ392470.1	NLD	2007	Mutation	Phylogenetics	Furin cleavage	FCoV
HQ392469.1	NLD	2008	Mutation	Phylogenetics	Furin cleavage	FCoV
HQ012372.1	NLD	2007	Mutation	Phylogenetics	Furin cleavage	FIPV
HQ012371.1	NLD	2008	Mutation	Phylogenetics	Furin cleavage	FCoV
HQ012370.1	NLD	2008	Mutation	Phylogenetics	Furin cleavage	FIPV
HQ012369.1	NLD	2007	Mutation	Phylogenetics	Furin cleavage	FIPV
HQ012368.1	NLD	2007	Mutation	Phylogenetics	/	FCoV
HQ012367.1	NLD	2007	Mutation	Phylogenetics	Furin cleavage	FIPV
GU553362.1	NLD	2007	/	Phylogenetics	Furin cleavage	FCoV
GU553361.1	NLD	2007	Mutation	Phylogenetics	Furin cleavage	FCoV
GQ152141.1	China/Taiwan	2007	Mutation	Phylogenetics	/	FCoV
FJ938062.1	NLD	2007	Mutation	Phylogenetics	Furin cleavage	FIPV
FJ938061.1	USA	1998	Mutation	Phylogenetics	Furin cleavage	FIPV
FJ938060.1	USA	1993	Mutation	Phylogenetics	Furin cleavage	FCoV
FJ938059.1	NLD	2007	Mutation	Phylogenetics	Furin cleavage	FCoV
FJ938058.1	NLD	2007	Mutation	Phylogenetics	Furin cleavage	FIPV
FJ938057.1	NLD	2007	/	Phylogenetics	Furin cleavage	FIPV
FJ938056.1	NLD	2007	Mutation	Phylogenetics	Furin cleavage	FIPV
FJ938055.1	NLD	2007	Mutation	Phylogenetics	Furin cleavage	FIPV
FJ938054.1	NLD	2007	Mutation	Phylogenetics	Furin cleavage	FIPV
FJ938053.1	NLD	2007	Mutation	Phylogenetics	Furin cleavage	FCoV
FJ938052.1	NLD	2007	Mutation	Phylogenetics	Furin cleavage	FCoV
FJ938051.1	USA	2002	Mutation	Phylogenetics	Furin cleavage	FCoV
EU186072.1	USA	1970s	Mutation	Phylogenetics	Furin cleavage	FIPV
DQ848678.1	UK	2006	Mutation	Phylogenetics	Furin cleavage	FIPV
DQ286389.1	USA	2005	Mutation	Phylogenetics	/	FIPV
DQ010921.1	USA	2005	/	Phylogenetics	/	FIPV
AY994055.1	USA	2008	Mutation	/	/	FIPV
